# A mixed methods evaluation of the large-scale implementation of a school- and community-based parenting program to reduce violence against children in Tanzania: a study protocol

**DOI:** 10.1186/s43058-021-00154-5

**Published:** 2021-05-20

**Authors:** Mackenzie Martin, Jamie Lachman, Joyce Wamoyi, Yulia Shenderovich, Mwita Wambura, Samwel Mgunga, Esther Ndyetabura, Amal Ally, Asheri Barankena, Amon Exavery, Nyasha Manjengenja

**Affiliations:** 1 Department of Social Policy and Intervention, University of Oxford, Oxford, UK; 2 MRC/CSO Social and Public Health Science Unit, University of Glasgow, Glasgow, UK; 3 National Institute for Medical Research, Mwanza, United Republic of Tanzania; 4 Wolfson Centre for Young People’s Mental Health, Cardiff University, Cardiff, UK; 5 Centre for the Development and Evaluation of Complex Interventions for Public Health Improvement (DECIPHer), School of Social Sciences, Cardiff University, Cardiff, UK; 6 Pact Tanzania, Dar es Salaam, United Republic of Tanzania; 7 Clowns Without Borders South Africa, Cape Town, South Africa

**Keywords:** Scale-up, Parenting, Implementation, Dissemination, Violence, Education, Fidelity, Families, Adolescents, Evaluation

## Abstract

**Background:**

Despite the rapid dissemination of parenting programs aiming to reduce and prevent violence against children (VAC) worldwide, there is limited knowledge about and evidence of the implementation of these programs at scale. This study addresses this gap by assessing the quality of delivery and impact of an evidence-based parenting program for parents/caregivers and their adolescent girls aged 9 to 14—Parenting for Lifelong Health Teens (PLH-Teens), known locally as Furaha Teens—on reducing VAC at scale in Tanzania. The study will explore participating family and staff perspectives on program implementation and examine factors associated with implementation and how implementation quality is associated with intervention outcomes when the program is delivered to approximately 50,000 parent-child dyads (*N* = 100,000) in schools and community centers across eight districts of Tanzania.

**Methods:**

This mixed-methods study will answer the following research questions: (1) what is the implementation quality and fidelity of PLH-Teens at scale in Tanzania; (2) what factors are associated with the quality of delivery and implementation fidelity of PLH-Teens; (3) how are implementation quality and fidelity associated with intervention outcomes; (4) what are participant and implementing staff perspectives on the acceptability, appropriateness, feasibility, benefits, and challenges of delivering PLH-Teens in their schools and communities; (5) what is the impact of PLH-Teens on VAC and participant well-being; and (6) how much does it cost to deliver PLH-Teens at scale? Qualitative and quantitative data will be collected directly from implementers, parents/caregivers, and adolescents using pre-post questionnaires, observational assessments, cost surveys, focus groups, and interviews. Qualitative data will be analyzed thematically with the aid of NVIVO software. Quantitative data will be cleaned and analyzed using methods such as correlation, regression, and structural equation models using Stata and R. COREQ and TREND guidelines will be used, where appropriate.

**Discussion:**

Findings will provide vital insights into some of the factors related to quality implementation at scale. Lessons learned regarding the implementation of PLH-Teens at scale will be applied in Tanzania, and also in the delivery of PLH parenting programs globally.

**Supplementary Information:**

The online version contains supplementary material available at 10.1186/s43058-021-00154-5.

Contributions to the literature

- The FAIR study responds to the urgent need to evaluate the implementation and impact of parenting programs that aim to prevent violence against children (VAC) at scale in low- and middle-income countries (LMICs) and in sub-Saharan Africa in particular.
- Despite emerging evidence on the effectiveness of parenting programs in LMICs, further research is necessary to understand the implementation, impact, optimization, and sustainability of these programs when delivered at scale.
- The FAIR study seeks to capitalize on innovative implementation science approaches to contribute to the evidence base in relation to PLH-Teens so as to maximize the prevention and reduction of VAC in Tanzania and in the 16 other LMICs where PLH-Teens is being implemented.

## Background

### Violence against children and parenting programs

Over one billion children experience violence each year with disproportionate numbers impacted in low- and middle-income countries (LMICs) [1, 2]. Violence has serious short- and long-term negative consequences for children, including for mental health, substance use, peer violence, delinquency, and the intergenerational transfer of violence (e.g., [3–8]). In Tanzania, over 72% of individuals aged 13–24 years old have experienced physical violence before age 18 [9]. Caregivers, other adult relatives, and teachers are the most commonly reported perpetrators of physical and emotional violence against children (VAC) in Tanzania, with corporal punishment considered normative [9]. As a Pathfinder Country, Tanzania has prioritized ending VAC and committed to reducing VAC by 50% by 2022 [10].

There is considerable evidence that parenting programs reduce VAC by improving parenting skills and reducing child behavior problems and by indirectly reducing associated risks such as youth violence, delinquency, and substance use as well as parental mental health difficulties (e.g., [11–18]). The potential of these programs has been recognized by international agencies, including the multi-agency *INSPIRE: Seven Strategies to End Violence Against Children* collection of evidence-informed approaches wherein parenting programs are recommended as a key strategy to prevent abuse [19].

### Implementation and scale-up of parenting programs

Given the encouraging evidence regarding the effectiveness of parenting programs aiming to reduce VAC in LMICs (e.g., [18]), there have been numerous calls to build the capacity of governments and agencies to implement such programs at scale (e.g., [20]). Scale-up may be defined as “deliberate efforts to increase the impact of health innovations successfully tested…so as to benefit more people and foster the development of sustainable policies and programs” [21]. However, there are numerous questions and challenges associated with scale-up, including whether such programs are perceived as being culturally acceptable and appropriate by beneficiaries and stakeholders, able to reach increased number of participants, feasible to deliver on a larger scale within existing delivery systems, delivered with fidelity to the program model, cost effective, and still effective when delivered beyond the scope of their original testing [21–24].

Research on family outcomes as part of the scale-up of parenting programs is limited, particularly in LMICs [25]. There are some studies in high-income countries (HICs) that have examined program impacts among entire populations. For instance, a study on the large-scale implementation of the Triple P program in North Carolina, USA, suggested some benefits in reducing child behavior problems and child maltreatment, even though a range of methodological challenges and limitations have been reported [25–27]. An evaluation of Triple P in Glasgow, UK, found no evidence of a population-level impact on child mental health [28]. There are other studies that have examined program impacts among large groups of participants. For instance, randomized controlled trials of the Nurse Family Partnership—a community-level home visiting program aiming to prevent child maltreatment by providing in-home support to low-income pregnant women and new-mothers—found the program to be effective in preventing child maltreatment and other outcomes in large samples [29, 30]. A study by Gray and colleagues examined the outcomes of various evidence-based parenting programs delivered on a large scale, including Triple P and Incredible Years, by comparing “service-led implementation” using data from 3706 families with previous “researcher-led” trials using data from 1390 families and found that community- and researcher-delivery resulted in similar outcomes suggesting that large-scale delivery is possible and effective for children and families [31].

In addition to a need for further research on outcomes, there is a need for more research on the implementation of parenting programs at scale to determine the extent and quality with which these programs are delivered [32]. Such research will then allow for an exploration of the impact of implementation at scale on program outcomes and the generation of insights regarding how programs might be improved [32]. Proctor’s taxonomy outlines eight implementation outcomes to examine to fully understand the quality of program implementation—adoption (the extent of program uptake), acceptability (participant satisfaction), appropriateness (program fit), feasibility (the extent to which the program can be delivered successfully, including consideration of its benefits and challenges), fidelity (adherence to the program theory and model), cost (time and resources required), penetration (the extent to which program delivery is embedded within existing services and systems), and sustainability (the practicality of long-term delivery) [22].

Several studies of parenting programs report on one or more of these implementation outcomes, including nascent insights emerging from studies in LMICs. To illustrate, a study on the Reach Up program in Brazil and Zimbabwe used qualitative methods to ascertain the perspectives of parents, facilitators, and supervisors on the program’s acceptability and appropriateness [33]. The authors of the paper drew insights about these implementation outcomes, including that parents were satisfied with the program. Other studies of parenting programs have explored the relationship between implementation outcomes and participant outcomes. For instance, a study on the implementation of the Growing Up Happily in the Family program in Spain explored a variety of implementation outcomes (including fidelity and acceptability) and analyzed whether they were associated with improvements in parental attitudes [34]. The researchers found that better fidelity and acceptability were associated with better parental attitudes. Similarly, a study on the Parent Management Training-Oregon (PMTO) program delivered at scale in Norway found that better facilitator delivery was correlated with improved parenting skills among program participants [35]. However, the majority of the evidence on implementation quality is from high-income countries. The Furaha Adolescent Implementation Research (or FAIR) study aims to help fill this gap by contributing knowledge regarding what implementation quality is like in a lower resource setting and at scale.

Other studies have examined factors that predict implementation outcomes. It has been recommended, for instance, that researchers explore the relationship between and the role of staff and organizations on implementation outcomes, including factors such as staff selection and training, ongoing monitoring and support of staff, and organizational leadership [32]. A study of a community-based intervention in South Africa and Malawi explored such a relationship; researchers looked at child outcomes in relation to whether implementing staff were paid or unpaid [36]. The study concluded that child outcomes were enhanced when the program was delivered by paid staff—an important finding given program delivery in LMICs leans towards volunteer-led delivery due to staffing shortages [36]. The FAIR study will add to the existing literature by examining staff and organizational factors, including facilitator characteristics such as the differences between teacher and volunteer delivery.

While there are some studies on the implementation and scale-up of parenting programs, the literature would benefit from enhanced evidence of family outcomes and quality of parenting program implementation at scale, how implementation outcomes are associated with participant outcomes, the factors that predict implementation outcomes, and how program implementation might be improved. The FAIR study aims to contribute in these areas by examining the factors, implementation, and outcomes of a parenting program delivered at scale in Tanzania.

### Parenting for Lifelong Health-Teens

Parenting for Lifelong Health (PLH) for Adolescents (PLH-Teens, known in Tanzania as Furaha Teens or “Happy Teens”) is among few low-cost parenting interventions for families with adolescents that have been rigorously tested in LMICs [37]. Originally developed and tested in South Africa, PLH-Teens is a parenting program rooted in social learning theory and behavior change principles that aims to reduce adolescent exposure to violence in the home and community by improving positive parenting and parent-child communication, while reducing familial conflict, harsh discipline, parenting stress, adolescent conduct problems, risky behavior, and mental ill health [38, 39]. Trained school and community facilitators engage parents/caregivers and adolescents in 14 weekly group sessions of approximately three hours in length using non-didactic, participatory methods including discussions, role-plays, problem-solving, and experiential activities [39]. As part of their participation, families receive incentives including meals and school supplies. Facilitators also assist families in developing child safety plans, responding to abuse, budgeting, and accessing medical and social services. Thus, PLH-Teens tackles a multitude of upstream and downstream contextual factors that lead to increased risk of VAC (e.g., [40–43]).

A recent cluster randomized trial in South Africa (*N* = 40 clusters, 552 parent/caregiver-adolescent dyads) found intervention effects for reduced abuse and corporal punishment as well as improved positive parenting, involvement, and monitoring based on caregiver reports at 5 to 9 months’ follow-up [37]. Effects on secondary outcomes included reductions in both adult and child substance use and parental stress, depression, endorsement of corporal punishment, and financial stress [37]. A cost-effectiveness analysis of PLH-Teens found that the intervention cost $972 USD per case of abuse prevented [44].

### PLH-Teens in Tanzania

Encouraging results from the cluster RCT [37] have contributed to the rapid dissemination of PLH-Teens in 16 countries to approximately 300,000 beneficiaries. Among these is the large-scale implementation of PLH-Teens in Tanzania that started in 2017 as part of the Kizazi Kipya (or “New Generation”) Project by Pact Tanzania. Kizazi Kipya is a USAID-PEPFAR-funded project aiming to enable more Tanzanian orphans and vulnerable children (OVC)—children, adolescents, and young people orphaned and made vulnerable by HIV and other adversities—to use age-appropriate HIV- and AIDS-related and other services for improved care, health, nutrition, education, protection, livelihoods, and psychosocial well-being. Through Kizazi Kipya, Pact Tanzania implements the DREAMS Initiative (Determined, Resilient, Empowered, AIDS-free, Mentored, and Safe) which aims to reduce HIV infection among adolescent girls and young women in HIV priority areas. As part of DREAMS, Pact is implementing the locally adapted and HIV-enhanced version of PLH-Teens, known as the Furaha Caring Families Program for Parents and Teens (Furaha Teens), for adolescent girls aged 9–14 and their parents/caregivers.

In 2020–2021, Pact is scaling-up PLH-Teens with 444 trained facilitators and 70 coaches to reach an additional 100,000 beneficiaries (approximately *N* = 50,000 adolescents and *N* = 50,000 parents/caregivers). The 2020–2021 delivery of PLH-Teens in Tanzania offers an unprecedented opportunity to examine the intervention and implementation outcomes when delivered at scale. As a result, this study—the Furaha Adolescent Implementation Research or FAIR study—will provide vital information on how to establish, implement, improve, and sustain high-quality delivery of PLH-Teens. The findings will also be of value to other parenting programs aiming to prevent VAC at scale.

### FAIR study

The FAIR study is linked to a larger study called the Scale-Up of Parenting Evaluation Research (SUPER) examining the implementation of PLH programs in multiple LMICs [45]. The SUPER study is using the Exploration, Preparation, Implementation and Sustainment (EPIS) framework to guide study questions and research tools as the framework has been widely used by practitioners and researchers to guide program implementation and evaluation [46]. EPIS has also been used to understand whether and how programs can be implemented successfully and sustainably in various settings on a large scale by considering four intervention phases—exploration, preparation, implementation, and sustainment [46–49]. The FAIR study is similarly rooted in the EPIS framework and is also informed by Proctor’s aforementioned taxonomy of implementation outcomes [22].

### Study aims and research questions

The FAIR study aims to examine the quality of implementation of PLH-Teens and its impact on preventing and reducing VAC at scale in Tanzania as well as consider factors associated with implementation and how implementation can be improved to optimize intervention impact. The study seeks to answer the following research questions: (1) what is the level of program implementation of PLH-Teens in terms of quality of delivery and implementation fidelity; (2) what factors are associated with the quality of delivery and implementation fidelity of PLH-Teens; (3) how are implementation quality and fidelity associated with intervention outcomes; (4) what are participant and implementing staff perspectives on the acceptability, appropriateness, feasibility, benefits, and challenges of delivering PLH-Teens in their schools and communities; (5) what is the impact of PLH-Teens on VAC and family well-being; and (6) how much does it cost to deliver PLH-Teens at scale?

## Methods

This mixed-methods study involves the integration of quantitative and qualitative methods to address the research questions. The data sources to be used are outlined in Table 1. Qualitative (including focus group discussions, in-depth interviews, and observation) and quantitative (merged secondary data collected via routine monitoring and evaluation by Pact Tanzania, local implementing partners or LIPs, and Clowns Without Borders South Africa or CWBSA) methods will be used to explore the impact, acceptability, appropriateness, feasibility, fidelity, and cost of PLH-Teens. As randomization to intervention and control groups is not possible, the study will make the most of the routine service delivery data available. Analyzing this data will allow for a unique inquiry into the real-world implementation of a parenting program at scale.

**Table 1:** Matrix of data collection methods

Type of data	Data collectors	Data collection method	Study participants
Primary data	FAIR research team	Focus group discussions	Adolescents
			Parents/caregivers
			Furaha facilitators and coaches
		In-depth interviews	Program coordinators and directors
			Pact Monitoring and Evaluation (M&E) team
			Furaha facilitators and coaches
			School principals
		Structured observations	Furaha Teens group sessions
			Furaha Teens coaching sessions
		Community of practice meeting	LIP and Pact staff
		Document review	All of the above
Secondary data	Pact Tanzania and LIPs (collected by Furaha facilitators) and other team members	Family reports of parenting practices, child behavior, child and caregiver mental health (routine data)	Parents/caregivers and adolescents
		Family enrolment, attendance, engagement, and dropout	
		Cost data	Facilitators, coaches, and LIP staff
		Surveys on the sociodemographic and professional background of facilitators and coaches delivering the program	Furaha facilitators and coaches
	CWBSA	Assessments of facilitator competent adherence	Furaha facilitators and coaches
		Assessments of coach delivery of facilitator supervision sessions	

### Collaborators and setting

The FAIR study is being conducted by the National Institute for Medical Research (NIMR) in Tanzania, the University of Oxford, CWBSA, and Pact Tanzania. The study will be conducted in eight districts of rural and semi-urban Tanzania: Kyela District Council (DC), Mbeya DC, Muleba DC, Shinyanga DC, Shinyanga Municipal Council, Kahama Town Council, Msalala DC, and Ushetu DC. PLH-Teens will be delivered by teachers in schools and in communities by volunteers (compensated with an honorarium) (*N* = 444) with Furaha program coaches (*N* = 70) providing facilitators with ongoing supervision. Facilitators will deliver the program via the coordination of five LIPs—Humuliza, Tadepa, Integrated Rural Development Organisation, Caritas, and Tanzania Red Cross Society.

### Study participants

The study will collect primary data from 48 program coaches, 96 program facilitators, 58 Pact Tanzania and LIP staff, eight school principals, three CWBSA staff, 155 parents/caregivers, and 155 adolescents. The study will also collect anonymized secondary data from approximately 50,000 parent-child dyads (*N* = 100,000), 444 program facilitators, 70 program coaches, and five LIPs. The inclusion criteria used to select study participants for primary and secondary data collection are outlined in Tables 2 and 3.

**Table 2:** Inclusion criteria for primary data study participants

Study participant group	Primary data inclusion criteria
Program Coaches (*N* = 70)	• Attended the Furaha Teens coach training workshop; and • Provided coaching to facilitators during the implementation of Furaha Teens.
Program Facilitators (*N* = 444)	• Teachers or community volunteers; • Attended the Furaha Teens facilitator training workshop; and • Implemented the Furaha Teens program.
Pact Tanzania and LIP Staff (*N* = 58)	• Staff member working for either Pact Tanzania or one of the LIPs delivering Furaha Teens.
School Principals (*N* = 8)	• Principal in a school where Furaha Teens was delivered.
CWBSA Staff (*N* = 3)	• Staff member working for CWBSA involved in the implementation or research associated with the FAIR Study.
Parents/caregivers (*N =* 155)	• Aged 18 or older; • Primary caregiver responsible for the care of an adolescent between the ages of 9 and 14 who attended the Furaha Teens program; and • Attended in the Furaha Teens program.
Adolescents (*N =* 155)	• Aged 9 to 14; • Consent provided by primary caregiver responsible for the adolescent’s well-being; • Assent provided by the adolescent; • Primary caregiver responsible for their care attended the Furaha Teens program; and • Attended in the Furaha Teens program.

**Table 3:** Inclusion for secondary data study participants

Study participant group	Secondary data inclusion criteria
Adolescents (*N =* 50,000)	• Adolescent girl aged 9 to 14; • Participated in the Kizazi Kipya Project; • In the same household as her parent/caregiver at least 4 days a week; • Parent/caregiver attended the Kizazi Kipya Project; • Consent provided by primary caregiver responsible for the adolescent’s well-being; and • Assent provided by the adolescent.
Parents/caregivers (*N =* 50,000)	• Aged 18 or older; • Primary caregiver responsible for the well-being and care of an adolescent girl between the ages of 9 and 14 who participated in the Kizazi Kipya Project; and • Attended the Kizazi Kipya Project.
Program Facilitators (*N* = 444)	• Attended a Furaha Teens facilitator training workshop; and • Facilitated Furaha Teens sessions.
Program Coaches (*N* = 70)	• Attended a Furaha Teens coach training workshop; and • Provided coaching to facilitators during the implementation of Furaha Teens.
LIPs (*N* = 5)	• Submitted a Request for Application (RFA) to the Kizazi Kipya Project to implement Furaha Teens in specific districts; and • Selected by Pact Tanzania to implement Furaha Teens.

### Study recruitment and informed consent

For the collection of primary qualitative data, purposive and snowball sampling will be used in collaboration with Pact Tanzania and LIPs to identify potential participants in each of the eight districts for semi-structured interviews and focus group discussions (FGDs). If potential participants consent to their contact details being shared with the researchers, the participants will be contacted by email or phone to outline the study prior to seeking informed consent. Alternatively, a researcher may be present during program training or another meeting to explain the study. Pact Tanzania staff will then provide potential participants with consent and assent forms (in the case of participants under age 18). Oxford and NIMR researchers will not be involved in recruiting participants for the secondary data. Instead, Pact Tanzania and CWBSA will ask all program participants if they would like to participate in the research upon their enrolment in Kizazi Kipya.

### Primary qualitative data collection

The qualitative data collection methods include semi-structured interviews and FGDs; structured observations of PLH-Teens group sessions conducted by facilitators; structured observations of facilitator supervision sessions conducted by coaches; analysis of policies, progress reports, and other documents anonymized and voluntarily provided by Pact Tanzania and CWBSA; and field notes taken by researchers during community of practice meetings with stakeholders. Qualitative data collection tools have been developed based on the EPIS framework and Proctor’s taxonomy. The interview, FGD, and observation guides cover relevant parts of the implementation process experienced by various participants (see Open Science Framework). For example, questions for facilitators focus on the implementation process since they are most familiar with implementation while questions for Pact managers emphasize exploration and sustainment.

#### Interviews and FGDs

Interviews will be conducted with coaches (*N* = 16), facilitators (*N* = 16), LIP staff (*N* = 8), school principals (*N* = 8), and CWBSA staff (*N* = 3). FGDs will be held with coaches (*N* = 32, 8/FGD), facilitators (*N* = 80, 10/FGD), parents/caregivers (*N* = 80, 10/FGD), and adolescents (*N* = 80, 10/FGD). All interviews (approximately 60–90 min) and FGDs (approximately 90–120 min) will be conducted in Kiswahili based on semi-structured guides (see Open Science Framework). The guides provide an outline of key topics and questions for the interviewers to ask study participants as well as leave room to delve into pertinent issues that emerge during interviews and FGDs. All interviews and FGDs will be audio-recorded with the permission of the participants. Where a participant declines, permission will be sought for field notes to be taken instead. Interview and FGD participants will be provided with lunch and transportation to and from the meeting venues (approximately $10–15 USD). In cases where face-to-face interviews and FGDs are not possible, interviews will be conducted remotely via telephone. While the importance of confidentiality will be emphasized during FGDs, participants will be informed about how limited researchers are in their ability to enforce post-discussion adherence to confidentiality commitments made by FGD participants.

#### Session observations

To better understand the implementation fidelity of PLH-Teens, researchers will conduct observations of program delivery and supervision sessions. Program participants and facilitators will be observed during program sessions (*N* = 5 sessions; 150 participants) and facilitators and coaches will be observed during supervision sessions (*N* = 5 sessions; 50 participants). The exact locations of the five program observations will be selected by the implementation team to take the variation and contextual factors of each district into consideration. A random selection of five coaching observations will be conducted in consultation with Pact Tanzania. Observations of program sessions and supervision sessions will follow structured observation guides (see Open Science Framework).

#### Document analysis

The researchers will conduct content analyses of Pact Tanzania and CWBSA reports to identify implementation barriers and supports as well as to determine how PLH-Teens fits within the larger Kizazi Kipya Project. Formal requests will be sent to partner organizations seeking permission to review and analyze relevant documents, with sensitive information redacted before the documents are shared and analyzed.

#### Community of practice meetings

Following program delivery, stakeholder engagement meetings will be held with government and non-government stakeholders involved in the implementation of PLH-Teens (*N* = 2 sessions; 50 participants). In these meetings, stakeholders will be asked to provide an overview of their experiences, including challenges implementing the program and possible solutions to the challenges identified. These participatory community of practice meetings will be held in Dar es Salaam during which researchers will take field notes.

### Secondary quantitative data collection

The study will analyze the following anonymized secondary process and outcome data from Pact Tanzania and CWBSA: pre-post surveys completed by parent/caregivers (*N* = 50,000); pre-post surveys completed by adolescents (*N* = 50,000); parent/caregiver and adolescent program attendance registers (*N* = 100,000 participants); facilitator demographic questionnaires (*N* = 444); coach demographic questionnaires (*N* = 70); coach assessments of facilitators (*N* = 444); CWBSA assessments of coaches (*N* = 70); LIP organizational surveys (*N* = 5); and implementation cost surveys (*N* = 300).

#### Family outcome and demographic measures

Pact Tanzania was provided with a set of process and outcome tools by CWBSA as part of the monitoring and evaluation technical support they provide to all implementing partners delivering PLH programs. CWBSA recommends and provides these tools because they are open-access and have been psychometrically tested in previous studies. Due to the large number of beneficiaries Pact Tanzania is reaching and their limited capacity to collect evaluation data, they are using abbreviated versions of the tools provided by CWBSA. An overview of these tools and their items is summarized in Additional file 8.

#### Implementation process measures

Pact Tanzania, LIPs, and CWBSA will collect data about parents/caregivers and adolescents (e.g., attendance), facilitators (e.g., demographic characteristics, fidelity), and coaches (e.g., demographic characteristics, fidelity) (see OSF page). The data will be used to understand the quality of program implementation, the factors that predict implementation outcomes, how implementation varies from context to context, and how implementation is associated with intervention outcomes. In particular, information about participant attendance, staff demographics, facilitator competent adherence, coach competent adherence, and organizational characteristics will be collected using a variety of measures (see Additional file 8). For example, data on facilitator competent adherence will be collected by Pact coaches using the PLH-Facilitator Assessment Tool for Teens (PLH-FAT-T)—an observational assessment tool administered by coaches based on live observations or video recordings of group sessions. Facilitator competent adherence is the skill with which a facilitator delivers intervention components and the strictness with which they follow the activities outlined in the program manual [50, 51]. All of the implementation data collected will be linked to parent/caregiver and adolescent outcomes through the use of unique identifiers supplied by LIPs, which will make it possible to link data from multiple sources. The data will be anonymized by the LIPs before it is shared with researchers.

#### Cost measures

Information about the time and resource costs of program set-up and implementation will be collected by Pact from facilitators, coaches, and LIP coordinators to determine how much program delivery costs at scale. Costing information will be collected using surveys which ask participants for retrospective estimates of the amount of time used or money expended on a program activity (see OSF page). The surveys were created based on resources provided by The Abdul Latif Jameel Poverty Action Lab. The collection of cost information will also include a review of program budgets, spending, and other data obtained from Pact Tanzania about the resources required to set-up and deliver the program.

### Data analysis

#### Qualitative analyses

Qualitative data will be transcribed verbatim and translated into English. Analysis will be conducted using NVIVO 12 qualitative analysis software. Multiple researchers will review a sample of the interview and FGD transcripts to generate a coding framework based on the research questions. Following the creation of the coding scheme, the data will be double coded to establish reliability among the researchers. Thereafter, data-driven coding will be used to identify concepts, relationships, and broad themes (thematic analysis). The findings will then be discussed by the research team to identify overarching themes and to select data segments that represent the key themes and divergent viewpoints. Where appropriate, COREQ standards will be used when reporting qualitative data [52].

#### Quantitative analyses

Quantitative data will be cleaned using Stata and analyzed in Stata and R using methods such as correlation and regression analyses, as well as structural equation models. The frequencies and distribution of each variable will be examined to check for any implausible values as well as to select the appropriate analysis method (e.g., a suitable regression link function). When there are more than two items in a given scale, coefficients such as Cronbach Alphas or Omegas will be used to assess the item-level reliability of the measures. Where possible, mixed effect models will be utilized to account for nesting within parenting groups [53]. Missing data will be addressed appropriately by considering the complete case observations as well as using full information maximum likelihood or multiple imputation, as appropriate [54, 55]. Where relevant, TREND guidelines will be used when reporting quantitative results [56].

#### Research question 1

The level of implementation of PLH-Teens delivery will be determined by analyzing data from family attendance registers; facilitator assessments; coach assessments; structured observations of group sessions; interviews held with facilitators, coaches, and LIP staff; and FGDs held with adolescents, parents/caregivers, facilitators, and coaches. Attendance rates and attendance trends among parents/caregivers and adolescents, as well as variations in attendance, and program completion rates will be calculated based on the attendance registers to determine the extent of participation in PLH-Teens. The level of competent adherence with which facilitators deliver the program will be determined using the results from the Facilitator Assessment Tool assessments completed by coaches. To examine the reliability and validity of the Facilitator Assessment Tool, a psychometric evaluation consisting of content validity (stakeholder perspectives from interviews and focus groups with facilitators, coaches, and CWBSA staff), intra-rater reliability (percentage agreements and intra-class correlations), inter-rater reliability (percentage agreements and intra-class correlations), internal consistency (Cronbach Alphas), construct validity (exploratory factor analyses), and predictive validity analyses will be performed. Similarly, the level of competent adherence with which coaches deliver facilitator supervision will be determined using the results from the Coach Assessment Tool assessments completed by CWBSA staff. Interviews, FGDs, and session observations will be used to expand upon and contextualize the findings regarding the demographic, attendance, facilitator competent adherence, and coach competent adherence data.

#### Research question 2

Factors associated with the quality of implementation will be examined using the socio-demographic data from the Facilitator and Coach Profile Forms; LIP organizational characteristics surveys; interviews; FGDs; and structured observations of group sessions. Correlation and regression analyses will be used to examine the relationship between facilitator and coach competent adherence and their associations with family, facilitator, coach, and organizational characteristics. Interviews, FGDs, and session observations will be used to expand upon and contextualize the findings.

#### Research question 3

A variety of data sources will be used to examine how implementation is associated with changes in VAC and family well-being. In particular, correlation and regression analyses will be used to look at whether pre-post changes in family outcomes are associated with family attendance, facilitator and coach competent adherence, and facilitator and coach characteristics, as well as LIP characteristics. Interviews, FGDs, and session observations will be used to expand upon and contextualize the findings.

#### Research question 4

Participant and implementing staff perspectives on the acceptability, appropriateness, feasibility, benefits, and challenges of delivering PLH-Teens in their communities will be examined by analyzing the interviews, FGDs, and session observations with school principals, facilitators, coaches, LIP staff, CWBSA staff, adolescents, and parents/caregivers.

#### Research question 5

Changes in VAC and participant well-being will be analyzed based on data gathered from parent/caregiver pre-post questionnaires, adolescent pre-post questionnaires, interviews, and FGDs. Multi-level models will be used to examine differences in pre- to post-intervention family-level outcomes and to compare differences in outcomes reported by both adolescents and parents/caregivers. Variation in the pre-post changes will be examined by participant baseline characteristics, and, if possible, by parenting group and LIP. The analyses will be similar to treatment-on-the-treated analyses since all participants included in the monitoring data would have engaged with the program to some extent. The levels of change reported by participants will be compared to the levels of change reported by the treatment and control groups in the randomized trial of the program in South Africa. Where possible, the reliability of the family survey items will also be examined using coefficients such as Cronbach Alphas or Omegas.

The findings from the interviews and FGDs  will also be analyzed to explore participant perspectives on the impacts of the program on them and their families. The interviews and FGDs will also reveal what impact implementing volunteers and staff assess the program had and will have on themselves, participants, schools, and communities.

#### Research question 6

The cost of delivering PLH-Teens at scale will be calculated using retrospective cost estimates provided by facilitators, coaches, and LIP coordinators and costing data provided by Pact Tanzania. Average costs will also be calculated and summarized for each program component (e.g., facilitator training, group sessions, supervision), family (parent/caregiver-adolescent dyad), district, and facilitator type (community volunteer or teacher).

A summary of the data that will be analyzed to answer each of the FAIR study’s six research questions is shown in Table 4.

**Table 4:** Evaluation matrix

Evaluation question	Data source
RQ1: What is the level of program implementation of PLH-Teens at scale in Tanzania in terms of quality of delivery and implementation fidelity?	1) Parenting for Lifelong Health-Facilitator Assessment Tool (PLH-FAT)—measures facilitator competence and adherence 2) Direct observation of group sessions using the structured observation guide 3) Semi-structured interviews held with facilitators, coordinators, coaches, and LIP staff 4) Focus group discussions (FGDs) held with adolescents, parents/caregivers, facilitators, and coaches
RQ2: What factors are associated with the quality of delivery and implementation fidelity of PLH-Teens?	1) PLH-FAT 2) Interviews 3) FGDs 4) Community of practice reflective meetings among LIPs, Pact, CWBSA and researchers 5) Facilitator Profile Form examining facilitator demographics including education level, experience, and professional background 6) Coach Profile Form examining coach demographics including education, experience, and professional background 7) LIP Organizational Characteristics Form 8) Direct observation of group sessions using the structured observation guide
RQ3: How are implementation quality and fidelity associated with intervention outcomes?	1) PLH-FAT 2) Interviews 3) FGDs 4) Community of practice reflective meetings 5) Facilitator Profile Form 6) Coach Profile Form 7) LIP Organizational Characteristics Form 8) Parent/caregiver- and adolescent-report on pre-post questionnaires 9) Parent/caregiver and adolescent program attendance data 10) Direct observation of group sessions using the structured observation guide
RQ4: What are participant and implementing staff perspectives on the acceptability, appropriateness, feasibility, benefits, and challenges of delivering PLH-Teens in their schools and communities?	1) Interviews with school principals, facilitators, coordinators, coaches, and LIP staff 2) FGDs with adolescents, parents/caregivers, facilitators, and coaches 3) Direct observation of group sessions using the structured observation guide
RQ5: What is the impact of PLH-Teens on VAC and participant well-being?	1) Parent/caregiver- and adolescent-report on pre-post questionnaires 2) Individual interviews with school principals, facilitators, coordinators, coaches, and LIP staff 3) FGDs with adolescents, parents/caregivers, facilitators, and coaches
RQ6: How much does it cost to deliver PLH-Teens at scale?	1) Facilitator cost surveys 2) Facilitator profile surveys 3) Coach cost surveys 4) LIP cost surveys

## Discussion

This mixed-methods implementation science study is a part of the first effort of its kind to examine the large-scale implementation of a parenting program aiming to reduce VAC in East Africa. The study’s results are important for the Parenting for Lifelong Health program and broader parenting program literature as they will provide key insights into the impact, acceptability, appropriateness, feasibility, costs, and optimization of large-scale parenting program delivery in both school and community settings. As the study will also examine factors associated with program implementation and outcomes, the results will help elucidate the potential mechanisms and processes through which program delivery and impacts can be improved in future (e.g., facilitator quality of delivery).

There are a number of practical, ethical, and operational issues that will require consideration during the study. These include safeguarding, potential implementation and funding delays, data quality concerns, staff burden issues, COVID-19-related impacts, and research uptake.

### Safeguarding

Since the study involves research with children and families who may be at risk of maltreatment and other adversities, research and implementation staff will comply with international standards concerning children’s rights to protection by following a Child Protection Protocol (see OSF page). All researchers interacting with minors will be trained on child protection and have a certificate on the protection of human research subjects. If sensitive information is to be collected, staff will be required to sign a confidentiality agreement. These expectations and procedures will be in addition to Pact and CWBSA’s existing policies and practices for child safeguarding.

### Implementation and funding delays

As program implementation is reliant on timely delivery of USAID funding to the prime implementing partner, spending/disbursement delays to LIPs could impact the study’s timeline. To accommodate potential delays, a flexible timeline has been adopted.

### Data quality and potential biases

Collecting comprehensive assessments of family-level outcomes from 100,000 beneficiaries is challenging. As a result, the study relies on existing data collection tools and processes developed and used during program implementation by Pact Tanzania’s Monitoring and Evaluation team. If timing allows, the research team will conduct random checks to verify the quality of implementation and outcome data. Among the observational facilitator and coach assessments, CWBSA will double code a random sample of program delivery videos to verify the accuracy and reliability of the assessments. As a significant amount of study data relies on self-reports, this data could be limited by recall and social desirability bias.

Where relevant, the study will take into consideration advice regarding how to manage and analyze data with FUPS characteristics (flawed, uncertain, proximate, and sparse), such as by presenting the data flow, conducting statistical sensitivity checks, and making the analyses accessible [57]. In the event of missing outcome data on individuals who are eligible but not included in the sample, sensitivity analysis will be done by replacing missing data with simulated data.

### Staff burden

To mitigate the possible overburdening of implementation staff tasked with both program delivery and data collection, the study focuses on answering research questions that respond to priority questions for implementers and draws on existing data collection tools and procedures to minimize the need for additional data collection.

### COVID-19 impacts

The delivery of PLH-Teens by Pact Tanzania was delayed for a number of months due to the COVID-19 pandemic. In response, the study approach and timeline have been adjusted. For instance, COVID-related questions were added to the primary qualitative data collection. Further, researchers are remaining flexible as the situation evolves. To illustrate, preparations will be made to conduct interviews via telephone, if necessary.

### Research uptake strategy

A variety of strategies will be employed to ensure that study findings are used to improve the implementation and scale-up of parenting programs aiming to reduce VAC in Tanzania and other LMICs. First, FAIR study researchers will collaborate and engage with key stakeholders involved in parenting programs to end VAC (e.g., policymakers, non-governmental organizations, and the Tanzanian Ministries of Education and Health). This engagement will involve meetings with key stakeholders to generate plans to put study findings into action such as by creating guidelines and policy briefs. Second, research findings will be used to enhance the future delivery of PLH-Teens by Pact Tanzania, CWBSA, LIPs, and global partners by hosting community of practice meetings, workshops, and training/capacity building sessions based on the findings. Third, keeping in mind that this research is ultimately for the benefit of children and families in Tanzania, findings will also be disseminated in user-friendly language to program participants through Pact’s community networks.

## Conclusion

The delivery of PLH-Teens in Tanzania to approximately 50,000 adolescent girls and their parents/caregivers (or 100,000 beneficiaries) represents an unprecedented opportunity to study the implementation and impact of a parenting program aiming to reduce VAC at scale in a LMIC. Although PLH-Teens has been delivered in 16 LMICs to over 300,000 beneficiaries, the Tanzanian delivery of PLH-Teens is the largest implementation of the program to date. To seize the opportunity to learn from the delivery of the program on such a large scale, this study plans to use innovative mixed-methods implementation science methods to examine the impact of PLH-Teens at scale and the key elements of program implementation identified by Proctor [22]—the acceptability, appropriateness, feasibility, benefits, and challenges of the intervention to families and implementation staff; the extent to which the program is adopted, implemented, and disseminated as intended; how implementation is associated with outcomes; the extent to which the program is embedded within existing systems and services; and how much it costs to deliver the program on a large-scale. The results will contribute to the larger SUPER study on the implementation of PLH programs globally [45]. The results will also be used to inform future thinking about the sustainability of the program and to communicate evidence-based recommendations regarding how program delivery could be modified so as to sustain and improve program effectiveness at scale both in Tanzania and 24 other LMICs where PLH is delivered.

## Supplementary Information

**Additional file 1.** TIDIeR checklist

**Additional file 2.** Oxford research ethics (R64777/RE001)

**Additional file 3.** NIMR research ethics (NIMR/HQ/R.8a/Vol.IX/3459)

**Additional file 4.** Pact research ethics (NIMR/HQ/R.8a/ Vol.IX/2902)

**Additional file 5.** SUPER research ethics (RE002 HEY BABY)

**Additional file 6.** Pact research ethics amendment/extension

**Additional file 7.** Letter of support from Evaluation Fund

**Additional file 8.** Secondary data collection measures

## Data Availability

Information and study materials are available on our Open Science Framework page: https://osf.io/m5fu2/. Intervention materials can be found on the World Health Organization website: https://www.who.int/teams/social-determinants-of-health/parenting-for-lifelong-health.

## Abbreviations

CWBSA: Clowns Without Borders South Africa
EPIS: Exploration, Preparation, Implementation and Sustainment framework
FAIR: The Furaha Adolescent Implementation Research Study
LIPs: Local implementing partners
LMICs: Low- and middle-income countries
NIMR: National Institute for Medical Research
PLH: Parenting for Lifelong Health
PLH-Teens: Parenting for Lifelong Health-Teens or locally known in Tanzania as Furaha Teens
VAC: Violence against children

## References

[1] Hillis S, Mercy J, Amobi A, Kress H (2016). Global prevalence of past-year violence against children: a systematic review and minimum estimates. Pediatrics..

[2] Stoltenborgh M, Bakermans-Kranenburg MJ, van Ijzendoorn MH (2013). The neglect of child neglect: a meta-analytic review of the prevalence of neglect. Soc Psychiatry Psychiatr Epidemiol.

[3] Font SA, Berger LM (2015). Child maltreatment and children’s developmental trajectories in early to middle childhood. Child Dev.

[4] Widom CS, Czaja SJ, Bentley T, Johnson MS (2012). A prospective investigation of physical health outcomes in abused and neglected children: new findings from a 30-year follow-up. Am J Public Health.

[5] Cowell RA, Cicchetti D, Rogosch FA, Toth SL (2015). Childhood maltreatment and its effect on neurocognitive functioning: timing and chronicity matter. Dev Psychopathol.

[6] Mills R, Alati R, Callaghan M, Najman JM, Williams GM, Bor W (2011). Child abuse and neglect and cognitive function at 14 years of age: findings from a birth cohort. Pediatrics..

[7] Moylan CA, Herrenkohl TI, Sousa C, Tajima EA, Herrenkohl RC, Russo MJ (2010). The effects of child abuse and exposure to domestic violence on adolescent internalizing and externalizing behavior problems. J Fam Violence.

[8] Walker SP, Wachs TD, Grantham-McGregor S, Black MM, Nelson CA, Huffman SL, et al. Inequality in early childhood: risk and protective factors for early child development. Lancet. 2011;378(9799):1325–38. 10.1016/S0140-6736(11)60555-2. 10.1016/S0140-6736(11)60555-2 21944375

[9] UNICEF (2009). Violence against children in Tanzania: findings from a national survey, 2009.

[10] UNICEF (2016). National Plan of Action to End Violence Against Women and Children in Tanzania 2017/8-2021/2.

[11] Thomas R, Zimmer-Gembeck MJ (2007). Behavioral outcomes of parent-child interaction therapy and Triple P—Positive Parenting Program: a review and meta-analysis. J Abnorm Child Psychol.

[12] Nowak C, Heinrichs N (2008). A comprehensive meta-analysis of Triple P-Positive Parenting Program using hierarchical linear modeling: effectiveness and moderating variables. Clin Child Fam Psychol Rev.

[13] Furlong M, McGilloway S, Bywater T, Hutchings J, Smith SM, Donnelly M (2012). Behavioural and cognitive-behavioural group-based parenting programmes for early-onset conduct problems in children aged 3 to 12 years. Campbell Syst Rev.

[14] Serketich WJ, Dumas JE (1996). The effectiveness of behavioral parent training to modify antisocial behavior in children: a meta-analysis. Behav Ther.

[15] Dretzke J, Davenport C, Frew E, Barlow J, Stewart-Brown S, Bayliss S, et al. The clinical effectiveness of different parenting programmes for children with conduct problems: a systematic review of randomised controlled trials. Child Adolesc Psychiatry Ment Health. 2009;3(1):7. 10.1186/1753-2000-3-7. 10.1186/1753-2000-3-7 PMC2660289 19261188

[16] Barlow J, Simkiss D, Stewart-Brown S (2006). Interventions to prevent or ameliorate child physical abuse and neglect: findings from a systematic review of reviews. J Child Serv.

[17] Chen M, Chan K (2016). Effects of parenting programs on child maltreatment prevention: a meta-analysis. Trauma Violence Abuse.

[18] Knerr W, Gardner F, Cluver L (2013). Improving positive parenting skills and reducing harsh and abusive parenting in low-and middle-income countries: a systematic review. Prev Sci.

[19] World Health Organization (2016). INSPIRE: Seven strategies for ending violence against children.

[20] Shenderovich Y, Lachman JM, Ward CL, Wessels I, Gardner F, Tomlinson M, et al. The science of scale for violence prevention: a new agenda for family strengthening in low- and middle-income countries. Front Public Health. 2021;9:581440. 10.3389/fpubh.2021.581440 PMC8044965 33869123

[21] Cash RA, editor. From one to many: scaling up health programs in low income countries; [collection of articles, International Conference on Scaling Up Health Programs, held in Dhaka, Bangladesh in December 2008]. University Press; 2011.

[22] Proctor E, Silmere H, Raghavan R, Hovmand P, Aarons G, Bunger A, et al. Outcomes for implementation research: conceptual distinctions, measurement challenges, and research agenda. Adm Policy Ment Health Ment Health Serv Res. 2011;38(2):65–76. 10.1007/s10488-010-0319-7. 10.1007/s10488-010-0319-7 PMC3068522 20957426

[23] World Health Organization. Nine steps for developing a scaling-up strategy. Geneva: WHO Press; 2010.

[24] Gottfredson DC, Cook TD, Gardner FE, Gorman-Smith D, Howe GW, Sandler IN (2015). Standards of evidence for efficacy, effectiveness, and scale-up research in prevention science: next generation. Prev Sci.

[25] Shapiro CJ, Prinz RJ, Sanders MR (2010). Population-based provider engagement in delivery of evidence-based parenting interventions: challenges and solutions. J Prim Prev.

[26] Eisner M (2014). The South Carolina Triple P System Population trial to prevent child maltreatment: seven reasons to be sceptical about the study results.

[27] Prinz RJ, Sanders MR (2007). Adopting a population-level approach to parenting and family support interventions. Clin Psychol Rev.

[28] Marryat L, Thompson L, Wilson P (2017). No evidence of whole population mental health impact of the Triple P parenting programme: findings from a routine dataset. BMC Pediatr.

[29] Olds DL (2006). The nurse-family partnership: an evidence-based preventive intervention. Infant Ment Health J.

[30] MacMillan HL, Wathen CN, Barlow J, Fergusson DM, Leventhal JM, Taussig HN (2009). Interventions to prevent child maltreatment and associated impairment. Lancet.

[31] Gray GR, Totsika V, Lindsay G (2018). Sustained effectiveness of evidence-based parenting programs after the research trial ends. Front Psychol.

[32] Tomlinson M, Hunt X, Rotheram-Borus MJ (2018). Diffusing and scaling evidence-based interventions: eight lessons for early child development from the implementation of perinatal home visiting in South Africa. Ann N Y Acad Sci.

[33] Smith JA, Baker-Henningham H, Brentani A, Mugweni R, Walker SP (2018). Implementation of Reach Up early childhood parenting program: acceptability, appropriateness, and feasibility in Brazil and Zimbabwe. Ann NY Acad Sci.

[34] Álvarez M, Rodrigo MJ, Byrne S (2018). What implementation components predict positive outcomes in a parenting program?. Res Soc Work Pract.

[35] Forgatch MS, DeGarmo DS (2011). Sustaining fidelity following the nationwide PMTO™ implementation in Norway. Prev Sci.

[36] Tomlinson M, Sherr L, Macedo A, Hunt X, Skeen S (2017). Paid staff or volunteers – does it make a difference? The impact of staffing on child outcomes for children attending community-based programmes in South Africa and Malawi. Glob Health Action.

[37] Cluver LD, Meinck F, Steinert JI, Shenderovich Y, Doubt J, Romero RH (2018). Parenting for lifelong health: a pragmatic cluster randomised controlled trial of a non-commercialised parenting programme for adolescents and their families in South Africa. BMJ Glob Health.

[38] Lachman JM. Building a rondavel of support: the development and pilot randomised controlled trial of a parenting programme to reduce the risk of child maltreatment in low-income families with children aged three to eight years in South Africa. Doctoral dissertation, University of Oxford; 2016.

[39] Wessels I, Ward CL (2015). A ‘best buy’for violence prevention: evaluating parenting skills programmes. S Afr Crime Q.

[40] Fraser MW (1997). Risk and resilience in childhood: an ecological perspective.

[41] Merrill LL, Hervig LK, Milner JS (1996). Childhood parenting experiences, intimate partner conflict resolution, and adult risk for child physical abuse. Child Abuse Negl.

[42] Garbarino J (1977). The human ecology of child maltreatment: a conceptual model for research. J Marriage Fam.

[43] Straus MA, Smith C (1990). Family patterns and child abuse. Physical violence in American families: Risk factors and adaptations to violence in 8,415 families.

[44] Redfern A, Cluver LD, Casale M, Steinert JI (2019). Cost and cost-effectiveness of a parenting programme to prevent violence against adolescents in South Africa. BMJ Glob Health.

[45] Shenderovich Y, Ward CL, Lachman JM, Wessels I, Sacolo-Gwebu H, Okop K, et al. Evaluating the dissemination and scale-up of two evidence-based parenting interventions to reduce violence against children: study protocol. Implementation Sci Commun. 2020;1(1):109. 10.1186/s43058-020-00086-6. 10.1186/s43058-020-00086-6 PMC7719848 38624613

[46] Moullin JC, Dickson KS, Stadnick NA, Rabin B, Aarons GA (2019). Systematic review of the exploration, preparation, implementation, sustainment (EPIS) framework. Implement Sci.

[47] Donenberg GR, Cohen MH, Ingabire C, Fabri M, Emerson E, Kendall AD, et al. Applying the Exploration Preparation Implementation Sustainment (EPIS) Framework to the Kigali Imbereheza Project for Rwandan Adolescents Living With HIV. J Acquir Immune Defic Syndr. 2019;82(3):S289–S98. 10.1097/QAI.0000000000002204. 10.1097/QAI.0000000000002204 PMC6884080 31764266

[48] Carcone AI, Coyle K, Gurung S, Cain D, Dilones RE, Jadwin-Cakmak L (2019). Implementation science research examining the integration of evidence-based practices into HIV prevention and clinical care: protocol for a mixed-methods study using the Exploration, Preparation, Implementation, and Sustainment (EPIS) Model. JMIR Res Protoc.

[49] Becan JE, Bartkowski JP, Knight DK, Wiley TR, DiClemente R, Ducharme L (2018). A model for rigorously applying the Exploration, Preparation, Implementation, Sustainment (EPIS) framework in the design and measurement of a large scale collaborative multi-site study. Health Justice.

[50] Fixsen DL, Naoom SF, Blase KA, Friedman RM, Wallace F, Burns B (2005). Implementation research: a synthesis of the literature.

[51] Forgatch MS, Patterson GR, DeGarmo DS (2005). Evaluating fidelity: predictive validity for a measure of competent adherence to the Oregon model of parent management training. Behav Ther.

[52] Tong A, Sainsbury P, Craig J (2007). Consolidated criteria for reporting qualitative research (COREQ): a 32-item checklist for interviews and focus groups. Int J Qual Health Care.

[53] Finch WH, Bolin JE, Kelley K. Multilevel modeling using R. Boca Raton, Florida: Crc Press; 2019. 10.1201/9781351062268.

[54] Schafer JL, Graham JW (2002). Missing data: our view of the state of the art. Psychol Methods.

[55] Muthén L (2010). Mplus Users Guide.

[56] Vandenbroucke JP, Von Elm E, Altman DG, Gøtzsche PC, Mulrow CD, Pocock SJ (2007). Strengthening the Reporting of Observational Studies in Epidemiology (STROBE): explanation and elaboration. PLoS Med.

[57] Wolpert M, Rutter H (2018). Using flawed, uncertain, proximate and sparse (FUPS) data in the context of complexity: learning from the case of child mental health. BMC Med.

