# Supplementary material for: A mixed methods evaluation of the large-scale implementation of a school- and community-based parenting program to reduce violence against children in Tanzania: a study protocol
Source: Implement Sci Commun. 2021 May 20;2:52. doi: 10.1186/s43058-021-00154-5 (PMC8136373; doi:10.1186/s43058-021-00154-5)
Supplement: Supplementary file 2 — Additional file 2. Oxford research ethics (R64777/RE001) [file 43058_2021_154_MOESM2_ESM.pdf]

SOCIAL SCIENCES & HUMANITIES INTERDIVISIONAL RESEARCH ETHICS COMMITTEE

Research Services, University of Oxford, Wellington Square, Oxford OX1 2JD
Tel: +44(0)1865 616576 Fax: +44(0)1865 280467
ethics@socsci.ox.ac.uk

UNIVERSITY OF OXFORD

11 September 2020

Dr Jamie Lachman
Department of Social Policy and Intervention

Dear Jamie

Research Ethics Approval (CUREC 2)
Ref No: R64777/RE001
Title: Furaha Adolescent Implementation Research (FAIR) Study

The above application has been considered on behalf of the Social Sciences and Humanities Interdivisional Research Ethics Committee (IDREC) in accordance with the procedures laid down by the University for ethical approval of all research involving human participants.

I am pleased to inform you that, on the basis of the information provided to the IDREC, the proposed research has been judged as meeting appropriate ethical standards, and accordingly approval has been granted.

Should there be any subsequent changes to the project that raise ethical issues not covered in the original application you should submit details to the IDREC for consideration: https://researchsupport.admin.ox.ac.uk/governance/ethics/apply/sshidrec#collapse394916.

Please note that your study may be selected for review by the SSH IDREC during an annual audit. You may also be required to submit a brief annual progress report on each anniversary of study approval, until the study is completed.

Yours sincerely,

JM Blaikie

Jennifer Blaikie
Research Ethics Manager

cc: Olivia Thornton
