# Supplementary material for: A mixed methods evaluation of the large-scale implementation of a school- and community-based parenting program to reduce violence against children in Tanzania: a study protocol
Source: Implement Sci Commun. 2021 May 20;2:52. doi: 10.1186/s43058-021-00154-5 (PMC8136373; doi:10.1186/s43058-021-00154-5)
Supplement: Supplementary file 3 — Additional file 3. NIMR research ethics (NIMR/HQ/R.8a/Vol.IX/3459) [file 43058_2021_154_MOESM3_ESM.pdf]

THE UNITED REPUBLIC OF TANZANIA

National Institute for Medical Research
3 Barack Obama Drive
P.O. Box 9653
11101 Dar es Salaam
Tel: 255 22 2121400
Fax: 255 22 2121360
E-mail: nimrethics@gmail.com

Ministry of Health, Community Development, Gender, Elderly & Children
University of Dodoma, College of Business Studies and Law
Building No. 11
P.O. Box 743
40478 Dodoma

NIMR/HQ/R.8a/Vol. IX/3459

30th June 2020

Dr Joyce Wamoyi
Principal Research Scientist
National Institute for Medical Research - Mwanza Centre
P O BOX 1462
Mwanza

RE: ETHICAL CLEARANCE CERTIFICATE FOR CONDUCTING MEDICAL RESEARCH IN TANZANIA

This is to certify that the research entitled: Furaha Adolescent Implementation Research Study (FAIR) (Wamoyi J. et al), has been granted ethical clearance to be conducted in Tanzania.

The Principal Investigator of the study must ensure that the following conditions are fulfilled:

1. Progress report is submitted to the Ministry of Health, Community Development, Gender, Elderly & Children and the National Institute for Medical Research, Regional and District Medical Officers after every six months.
2. Permission to publish the results is obtained from National Institute for Medical Research.
3. Copies of final publications are made available to the Ministry of Health, Community Development, Gender, Elderly & Children and the National Institute for Medical Research.
4. Any researcher, who contravenes or fails to comply with these conditions, shall be guilty of an offence and shall be liable on conviction to a fine as per NIMR Act No. 23 of 1979, PART III Section 10(2).
5. Sites: Kagera, Mbeya and Shinyanga regions.

Approval is valid for one year. 30th June 2020 to 29th June 2021.

Name: Prof. Yunus Daud Mgaya

Signature
CHAIRPERSON
MEDICAL RESEARCH COORDINATING COMMITTEE

Name: Prof. Abel Nkono Makubi

Signature
CHIEF MEDICAL OFFICER
MINISTRY OF HEALTH, COMMUNITY DEVELOPMENT, GENDER, ELDERLY & CHILDREN

CC: Director, Health Services-TAMISEMI, Dodoma
RMO of Kagera, Mbeya and Shinyanga regions.
DMO/DED of respective districts.
