# Supplementary material for: A mixed methods evaluation of the large-scale implementation of a school- and community-based parenting program to reduce violence against children in Tanzania: a study protocol
Source: Implement Sci Commun. 2021 May 20;2:52. doi: 10.1186/s43058-021-00154-5 (PMC8136373; doi:10.1186/s43058-021-00154-5)
Supplement: Supplementary file 4 — Additional file 4. Pact research ethics (NIMR/HQ/R.8a/ Vol.IX/2902) [file 43058_2021_154_MOESM4_ESM.pdf]

THE UNITED REPUBLIC OF TANZANIA

National Institute for Medical Research
3 Barack Obama Drive
P.O. Box 965
11101 Dar es Salaam
Tel: 255 22 2121400
Fax: 255 22 2121360
E-mail: nimrethics@gmail.com

Permanent Secretary (Health)
Ministry of Health, Community Development, Gender, Elderly and Children
Government City Mtumba, Health Road
P.O. Box 743
40478 Dodoma

NIMR/HQ/R.8c/Vol. I/876

16th December, 2020

Dr. Joyce Wamoyi
Principal Research Scientist
National Institute for Medical Research – Mwanza Centre
P.O.BOX 1462
Mwanza.

RE: ETHICAL APPROVAL FOR PROTOCOL AMENDMENT

This letter is to confirm that your application for amendment of a protocol on the study entitled: Furaha adolescent implementation research study (FAIR) (Wamoyi J. et al) with ref. NIMR/HQ/R.8a/Vol. IX/3459, dated 30th June, 2020 has been granted ethical clearance to be conducted in Tanzania.

Approval is for the following amendments:

1. Addition of the following documents to the protocol:
   - Cost data collection tool
   - Research team safety protocol
   - Supervision session observation form
   - Coach and facilitator assessment survey tools (2)
   - Facilitator demographic survey
   - Group attendance register

Approval is valid until 29th June, 2021.

Name: Prof. Yunus Daud Mgaya

Signature
CHAIRPERSON
MEDICAL RESEARCH
COORDINATING COMMITTEE

Name: Prof. Abel Nkono Makubi

Signature
CHIEF MEDICAL OFFICER
MINISTRY OF HEALTH, COMMUNITY DEVELOPMENT, GENDER, ELDERLY & CHILDREN
