# Supplementary material for: A mixed methods evaluation of the large-scale implementation of a school- and community-based parenting program to reduce violence against children in Tanzania: a study protocol
Source: Implement Sci Commun. 2021 May 20;2:52. doi: 10.1186/s43058-021-00154-5 (PMC8136373; doi:10.1186/s43058-021-00154-5)
Supplement: Supplementary file 5 — Additional file 5. SUPER research ethics (RE002 HEY BABY) [file 43058_2021_154_MOESM5_ESM.pdf]

Department of Social Policy and Intervention
University of Oxford

Barnett House, 32 Wellington Square,
Oxford, OX1 2ER, United Kingdom
www.spi.ox.ac.uk

Professor Lucie Cluver
Department of Social Policy and Intervention
University of Oxford

SPICUREC1a__20_015

30th July 2020

Dear Lucie,

Parenting for Lifelong Health: Scale-Up of Parenting Evaluation Research (PLH-SUPER)

Your application for research ethics approval in connection with your research project has been considered by the Departmental Research Ethics Committee (DREC) in accordance with the procedures laid down by the University for Ethical Approval.

I am pleased to inform you that, on the basis of the information provided, the proposed research has been judged as meeting appropriate ethical standards and DREC approval has been granted.

If any revisions to your research methodology are made subsequent to this approval, these must be detailed in writing and submitted to DREC immediately.

Yours sincerely,

Jane Barlow

Professor Jane Barlow
Research Director and Acting Chair of DREC
