# Supplementary material for: A mixed methods evaluation of the large-scale implementation of a school- and community-based parenting program to reduce violence against children in Tanzania: a study protocol
Source: Implement Sci Commun. 2021 May 20;2:52. doi: 10.1186/s43058-021-00154-5 (PMC8136373; doi:10.1186/s43058-021-00154-5)
Supplement: Supplementary file 6 — Additional file 6. Pact research ethics amendment/extension [file 43058_2021_154_MOESM6_ESM.pdf]

THE UNITED REPUBLIC OF TANZANIA

National Institute for Medical Research
3 Barack Obama Drive
P.O. Box 9653
11101 Dar es Salaam
Tel: 255 22 2121400
Fax: 255 22 2121360
Email: nimrethics@gmail.com

Permanent Secretary (Health)
Ministry of Health, Community
Development, Gender, Elderly & Children
Government City Mtumba, Health Road
P.O. Box 743
40478 Dodoma

NIMR/HQ/R.8c/Vol. I /1634

11th December, 2020

Dr. Amon Exavery
Pact Tanzania
C/o Dr. Naftali Ng'ondi
MoHCDGEC
P O Box 32652
Dar es Salaam

RE: APPROVAL FOR EXTENSION OF ETHICAL CLEARANCE

This letter is to confirm that your application for extension on the already approved proposal: Inspiring an evidence-base for parenting programmes to end child abuse: including Tanzania data in a multi-country secondary analysis (Exavery A. et al) whose local investigator is Dr. Naftali Ng'ondi of MoHCDGEC, has been approved.

The extension approval is based on the progress report dated 18th December, 2020 on the project, Ref. NIMR/HQ/R.8a/Vol. IX/2902, dated 03rd October, 2018. Extension approval is valid until 02nd October, 2021.

The Principal Investigator must ensure that other conditions of approval remain as per ethical clearance letter. The PI should ensure that progress and final reports are submitted in a timely manner.

Name: Prof. Yunus Daud Mgaya

Signature
CHAIRPERSON
MEDICAL RESEARCH
COORDINATING COMMITTEE

Name: Prof. Abel Nkono Makubi

Signature
CHIEF MEDICAL OFFICER
MINISTRY OF HEALTH, COMMUNITY
DEVELOPMENT, GENDER, ELDERLY
& CHILDREN
