# Supplementary material for: A mixed methods evaluation of the large-scale implementation of a school- and community-based parenting program to reduce violence against children in Tanzania: a study protocol
Source: Implement Sci Commun. 2021 May 20;2:52. doi: 10.1186/s43058-021-00154-5 (PMC8136373; doi:10.1186/s43058-021-00154-5)
Supplement: Supplementary file 8 — Additional file 8. Secondary data collection measures [file 43058_2021_154_MOESM8_ESM.docx]

**Secondary Data Collection Measures**

**Family Outcome and Demographic Measures**

**Demographic items.**

The demographic information that will be collected includes parent/caregiver and adolescent age, gender, education level, economic status, food security, health insurance status, HIV status, and home-level risk factors of VAC (15 items).

**Positive parenting.**

An adapted version of the Alabama Parenting Questionnaire (APQ) (Frick, 1991) will be used to assess parent/caregiver- and child-reports on the frequency of specific parent/caregiver behaviors towards adolescents in the past month on a seven-point Likert scale (0 = *never*; 6 = *always*). The APQ measures parental involvement (3 items, e.g., “you/your caregiver get(s) involved in activities that your child/you like(s)”) and parental monitoring (3 items, e.g., “you/your child are/is left at home without adult supervision”) subscales. Items are summed to create a total positive parenting score as well as for each subscale.

**Child behavior and mental health.**

The Strengths and Difficulties Questionnaire (SDQ) (Goodman, 1997) will be used to assess child behavior problems. The tool asks parents/caregivers and adolescents to indicate the frequency of specific child behaviors using a three-point Likert scale (1 = *not true*; 3 = *very true*). Pact Tanzania uses the SDQ Conduct Problems subscale to assess externalizing behavior (5 items, e.g., “I get/your child gets angry and often lose(s) my/their temper”) and the SDQ Emotional Problems subscale to assess internalizing behavior (5 items, e.g., “I am/your child is often unhappy, downhearted or tearful”). The items in each subscale are summed, with higher scores indicating more behavioral problems.

**Child maltreatment.**

The ISPCAN Child Abuse Screening Tools-Trial Version (ICAST-T) will be used to assess parent/caregiver- and child-reports on child maltreatment (4 items). The tool asks parents/caregivers and adolescents to indicate the frequency of emotional abuse (e.g., “shouting or screaming” and “saying mean things to upset,”) and physical abuse (e.g., “spanking, slapping, or hitting with a hand” and “discipline with an object like a stick or belt,”) over the past month using a nine-point Likert scale (0 = *never*; 8 = *8 or more times*) (Meinck et al., 2018). Items are summed to create a total child maltreatment score as well as a score for each subscale.

**Acceptability of corporal punishment***.*

One item from the Multiple Indicator Cluster Survey (MICS) will be used to assess parents/caregivers and adolescent views on the acceptability of corporal punishment. This item asks respondents to indicate the extent to which they agree or disagree (1 = *strongly disagree*; 5 = *strongly agree*) with the statement: “In order to bring up, raise, or educate a child properly, a child needs to be physically punished.”

**Parental depression.**

Parental depression will be assessed using the Centre for Epidemiologic Studies Depression Scale (CES-D 10) (Irwin et al., 1999). The tool asks parents/caregivers to respond to items related to how they have felt over the past seven days (3 items, e.g., “How often in the past week have you felt depressed?”). Responses are coded on a four-point Likert scale (1 = *rarely or none of the time;* 4 = *most or all of the time*). Items are summed with higher scores indicating higher levels of parental depression.

**Parental support of education.**

An adapted version of the Parental Support for School Scale (Ceballo et al., 2014) will be used to measure parent/caregiver- and adolescent-reports on the frequency of supportive behavior by parents/caregivers towards their children’s learning (e.g. “I/your caregiver support(s) my child’s/your schoolwork in any way that I/they can” and “I/your caregiver praise(s) my child/you for working hard at school”) using a five point Likert scale (1 = *never; 5 = always*). Items are summed to create a frequency score, with higher scores suggesting more parental support and value for school.

**Economic strengthening.**

The Family Financial Coping Scale (FFCS; 6 items) will be used to gain insight into the financial status of the participating families. The tool asks parents/caregivers to respond to items related to financial matters in the past month. These items include questions on whether parents/caregivers were worried about money, saved money, and ran out of money to buy certain items, such as two meals a day.

**Intimate partner violence.**

Parent/caregiver reports of intimate partner violence victimization and perpetration in the past month will be assessed using four items adapted from the Revised Conflict Tactics Scale Short Form (CTS2S; 8 items) (Straus et al., 1996). Items included in the tool ask about the frequency of physical assault (e.g., “my partner/I hit, push, shove, or slap me/my partner”) and psychological aggression (e.g., “my partner/I insult(s), shout(s), yell(s) or swear(s) at me/them”). Answers are coded using the same nine-point Likert scale as the ICAST (0 = *never*; 8 = *8 or more times*). Items are summed, with higher scores indicating higher levels of victimization or perpetration of intimate partner violence.

**School violence.**

Child experience of school violence will be assessed using three items, one on bullying (“In the past 4 weeks, how often did you experience any bullying at school such as persistent name calling, threats of violence, or physical attacks?”), one on physical discipline from adults at school (“In the past 4 weeks, how often did a teacher or any other adult discipline you at school by hitting you with their hand or an object like a stick or belt?”), and one on verbal discipline from adults at the school (“In the past 4 weeks, how often did a teacher or other adult at your school discipline you by shouting, yelling, or screaming at you?”). These questions were designed by FAIR study researchers and will be coded using the same nine-point Likert scale as the ICAST (0 = *never*; 8 = *8 or more times*). Items are summed with higher scores indicating higher levels of school violence victimization.

**Other family-level measures.**

As part of their monitoring and evaluation of the broader Kizazi Kipya Project, Pact Tanzania collects a variety of information from all enrolled families: HIV status and HIV risk assessment; caregiver-child communication on sexual and reproductive health (about puberty and growth, safe sex practices and contraceptive methods, relationship with adults, sugar daddy/sugar mummy); food security; and other sociodemographic indicators such as wealth quintile and household size.

**Implementation Process Measures**

**Attendance.**

Attendance refers to the number of sessions attended by a program participant out of the total possible number of sessions offered to the participant. Attendance data will be collected by Pact Tanzania via attendance registers completed by facilitators each week. An overall attendance rate will be calculated for each parent/caregiver-child dyad.

**Staff demographic data.**

Pact will collect demographic data on facilitators and coaches using an implementation staff questionnaire (Facilitator and Coach Profile Forms). The demographic data to be collected includes facilitator/coach age, gender, marital status, parental status, number and age of children, employment status, and educational level. The questionnaires will also assess facilitator/coach self-efficacy and their view on the acceptability of corporal punishment.

**Facilitator Competent Adherence**

Data on facilitator competent adherence will be collected by Pact coaches using the PLH-Facilitator Assessment Tool for Teens (PLH-FAT-T) - an observational assessment tool administered by coaches based on live observations or video recordings of group sessions. The PLH-FAT-T was developed by the study investigators and PLH program developers to assess the proficiency of program delivery by facilitators as a prerequisite to their certification. The items in the tool are grouped into two subscales based on the core activities and process skills required of facilitators. The assessment of core activities (22 items) requires coaches to rate the quality of facilitator delivery during home activity discussions (11 items) and role-plays (11 items). The assessment of process skills (28 items) requires coaches to rate the quality of facilitator use of modelling skills (5 items), the Accept-Explore-Connect-Practice facilitation technique (8 items), and collaborative leadership skills (15 items). Each item is rated on a three-point Likert scale ranging from zero to two (0= *inadequate*, 1= *good*, 2= *excellent*). By totaling the score from all items, an overall impression score is produced and represented as a percentage.

**Coach competent adherence.**

Data on coach competent adherence will be collected by CWBSA staff using the PLH-Coach Assessment Tool (PLH-CAT) which is an observational assessment tool similar to the PLH-FAT-T. The PLH-CAT assesses the quality of coaching provided to facilitators based on either live observations or video recordings of coaching sessions. The tool includes an activity subscale which assesses a coach’s review of delivery highlights and challenges (12 items) and use of process skills similar to those assessed by the PLH-FAT-T (26 items). Each PLH-CAT item is rated on a three-point Likert scale ranging from zero to two (0= *inadequate*, 1= *good*, 2= *excellent*). By totaling the score from all items, an overall impression score is produced and represented as a percentage.

**Organizational surveys.**

A short organizational survey has been developed to gather LIP characteristics from staff and to explore their observations about variations in program adoption and differences between the districts.

**References**

Ceballo, R., Maurizi, L. K., Suarez, G. A., & Aretakis, M. T. (2014). Gift and sacrifice: Parental involvement in Latino adolescents’ education. *Cultural Diversity and Ethnic Minority Psychology, 20*(1), 116.

Frick, P. J. (1991). The Alabama parenting questionnaire. *Unpublished rating scale, University of Alabama*.

Goodman, R. (1997). The Strengths and Difficulties Questionnaire: a research note. *Journal of Child Psychology and Psychiatry, 38*(5), 581-586.

Irwin, M., Artin, K. H., & Oxman, M. N. (1999). Screening for depression in the older adult: criterion validity of the 10-item Center for Epidemiological Studies Depression Scale (CES-D). *Archives of internal medicine, 159*(15), 1701-1704.

Meinck, F., Boyes, M. E., Cluver, L., Ward, C. L., Schmidt, P., DeStone, S., & Dunne, M. P. (2018). Adaptation and psychometric properties of the ISPCAN Child Abuse Screening Tool for use in trials (ICAST-Trial) among South African adolescents and their primary caregivers. *Child Abuse & Neglect, 82*, 45-58.

Straus, M. A., Hamby, S. L., Boney-McCoy, S., & Sugarman, D. B. (1996). The revised conflict tactics scales (CTS2) development and preliminary psychometric data. *Journal of family issues, 17*(3), 283-316.
