# Supplementary material for: A mixed methods evaluation of the large-scale implementation of a school- and community-based parenting program to reduce violence against children in Tanzania: a study protocol
Source: Implement Sci Commun. 2021 May 20;2:52. doi: 10.1186/s43058-021-00154-5 (PMC8136373; doi:10.1186/s43058-021-00154-5)
Supplement: Supplementary file 7 — Additional file 7. Letter of support from Evaluation Fund [file 43058_2021_154_MOESM7_ESM.pdf]

Network of European Foundations

The Evaluation Fund
Reducing Violence Against Children

Dr. Joyce Wamoyi
National Institute of Medical Research,
PO Box 1462
Mwanza, Tanzania.

24th February 2020

Ref: EF-2019-1107

Dear Dr. Wamoyi,

Re: Approval of Application to the Evaluation Fund's Call for Proposals 2019

Thank you for submitting a full research proposal to the Evaluation Fund. We are pleased to inform you that your Proposal has been selected for the award of a grant of **$199,967.** Attached is a grant agreement outlining the details and conditions of this award. Please read this document carefully to ensure that all information and conditions therein, (including the proposal and budget annexes), are accurate and acceptable to you. Complete the necessary organisational bank details section. The agreement should then be signed by the legally authorised person in your organisation. At your earliest convenience, the signed copy should be scanned and emailed to NEF, along with the completed request for the first payment of **$119,980** (60%), in the attached format. If you have any queries, please do not hesitate to contact **The Evaluation Fund Program Officer, Giulia Barnhisel**. Congratulations on your successful application and we look forward to working with you on this exciting project.

Yours Sincerely,

Peggy Saïller

NEF Executive Director

Attachments:

1. *Grant Agreement with approved Proposal & Budget*
2. *Payment Request Template*
